# Imbalance of Functional Connectivity and Temporal Entropy in Resting-State Networks in Autism Spectrum Disorder: A Machine Learning Approach

**DOI:** 10.3389/fnins.2018.00869

**Published:** 2018-11-27

**Authors:** Robert X. Smith, Kay Jann, Mirella Dapretto, Danny J. J. Wang

**Affiliations:** ^1^NeuroImaging Laboratories (NIL) at Washington University School of Medicine, Washington University in Saint Louis, Saint Louis, MO, United States; ^2^Keck School of Medicine of USC, University of Southern California, Los Angeles, CA, United States; ^3^Department of Psychiatry and Biobehavioral Sciences, University of California Los Angeles, Los Angeles, CA, United States

**Keywords:** complexity, resting-state, fMRI, connectivity, dynamics, Autism Spectrum Disorders

## Abstract

**Background:** Two approaches to understanding the etiology of neurodevelopmental disorders such as Autism Spectrum Disorder (ASD) involve network level functional connectivity (FC) and the dynamics of neuronal signaling. The former approach has revealed both increased and decreased FC in individuals with ASD. The latter approach has found high frequency EEG oscillations and higher levels of epilepsy in children with ASD. Together, these findings have led to the hypothesis that atypical excitatory-inhibitory neural signaling may lead to imbalanced association pathways. However, simultaneously reconciling local temporal dynamics with network scale spatial connectivity remains a difficult task and thus empirical support for this hypothesis is lacking.

**Methods:** We seek to fill this gap by combining two powerful resting-state functional MRI (rs-fMRI) methods—functional connectivity (FC) and wavelet-based regularity analysis. Wavelet-based regularity analysis is an entropy measure of the local rs-fMRI time series signal. We examined the relationship between the RSN entropy and integrity in individuals with ASD and controls from the Autism Brain Imaging Data Exchange (ABIDE) cohort using a putative set of 264 functional brain regions-of-interest (ROI).

**Results:** We observed that an imbalance in intra- and inter-network FC across 11 RSNs in ASD individuals (*p* = 0.002) corresponds to a weakened relationship with RSN temporal entropy (*p* = 0.02). Further, we observed that an estimated RSN entropy model significantly distinguished ASD from controls (*p* = 0.01) and was associated with level of ASD symptom severity (*p* = 0.003).

**Conclusions:** Imbalanced brain connectivity and dynamics at the network level coincides with their decoupling in ASD. The association with ASD symptom severity presents entropy as a potential biomarker.

## Introduction

Autism spectrum disorder (ASD) impacts the neurodevelopment of networks underlying social function and communication as well as sensorimotor abilities (APA, 1994). ASD has been linked to imbalanced functional connectivity (FC) in the brain (Jeste, 2011). FC measures synchronous neuronal signaling and has been used to identify several resting-state networks (RSNs) (Greicius et al., 2004; Seeley et al., 2009). Studies have reported intra- and inter-network FC among several RSNs to be either reduced (Villalobos et al., 2005; Welchew et al., 2005; Kana et al., 2006, 2007; Kleinhans et al., 2008; Uddin et al., 2013) or increased (Anderson et al., 2011; Supekar et al., 2013; Uddin et al., 2013) in ASD. Most work has focused on intra-network FC for specific RSNs. Notably, several studies have reported that the Default Mode Network (DMN), a set of brain regions that exhibit increased activity in the absence of an external stimuli (Raichle and MacLeod, 2001), exhibits both increased and decreased FC in ASD (Jann et al., 2015). However, research focused on a specific network is inherently limited at delineating the mechanisms of brain disruption at the global level. A growing number of reports have also shown that inter-network FC is also strongly impacted in ASD (Belmonte et al., 2004; Courchesne et al., 2007; Rudie et al., 2013; Cerliani et al., 2015).

Imbalance of excitation and inhibition within neural microcircuitry may impair the formation of intra- and inter-network connections that typify the segregation of RSNs during typical neurodevelopment. Hyper-excitability (elevated excitation/inhibition balance) has been hypothesized (E/I hypothesis) as an underlying mechanism for behavioral deficits in ASD (Rubenstein and Merzenich, 2003; Chao et al., 2010; Vattikuti and Chow, 2010; Yizhar et al., 2011). However, reconciling cortical dynamics with spatial network connectivity remains a difficult task. Resting-state functional MRI (rs-fMRI) is a widely used method offering a balance between temporal and spatial resolution. The rs-fMRI time series signal represents intrinsic blood oxygen level dependent (BOLD) activity that is correlated with neuronal activation (Logothetis et al., 2001). Evidence of spontaneous BOLD fluctuations suggests that stochastic processes govern neuronal activity (He et al., 2010). However, most studies investigate brain connectivity using FC analysis (e.g., mean intra- and inter-network correlations) which carries little information about the dynamic structure typifying neuronal activity. The relationship between FC and brain dynamics in ASD is not well-understood.

Recently, non-linear statistical measures based on approximate entropy (Pincus, 1991) and sample entropy (Richman and Moorman, 2000; Costa et al., 2002) have been used to investigate the dynamic structure and complexity of the brain by characterizing the recurring patterns of temporal fluctuations (Smith et al., 2014). A time series containing many repetitive patterns has relatively small entropy. Conversely, a time series containing few repetitive patterns has a higher entropy. Entropy studies have shown changes in dynamics in aging (Liu et al., 2012; Yang et al., 2013a), Alzheimer's disease (Yang et al., 2013b), schizophrenia (Takahashi et al., 2010), and depression (Pei-Shan Ho et al., 2018). Here we investigate the relationship between FC and brain dynamics at the network level using a recently developed wavelet-based regularity analysis (Smith et al., 2015). This approach to assess network dynamics is based on noise estimation capabilities of the wavelet transform to measure recurrent temporal pattern stability within the rs-fMRI signal across multiple temporal scales. The method consists of performing a stationary wavelet transform (SWT) to preserve signal structure, followed by construction of “lagged” subsequences to adjust for correlated features, and finally the calculation of sample entropy across wavelet scales based on an “objective” estimate of noise level at each scale.

Previous applications of wavelet-based regularity analysis showed the DMN, the most ‘active' areas of the brain at rest (De Luca et al., 2006), exhibited higher rs-fMRI signal entropy than rest of the brain (Smith et al., 2015). This suggested increased rs-fMRI signal activity is characterized by not only increased amplitudes, but also more complex trajectories through a diverse array of temporal patterns. Further investigation of wavelet-based regularity suggested it may be sensitive to neurobiological changes that underscore cognitive dysfunction. Specifically, widespread entropy differences in the DMN and executive control networks were detected between individuals with mild cognitive impairment and healthy controls. Taken together, these observations suggest wavelet-based regularity analysis is a promising measure of the rs-fMRI signal's dynamic structure.

Leveraging the spatial resolution of rs-MRI, we use machine learning to model the FC-entropy relationship across cortical and subcortical RSNs. We hypothesized that FC measures would be associated with RSN entropy in both ASD and TD participants. However, per the E/I hypothesis, we expected the FC-entropy relationship to be significantly weaker in ASD participants.

## Methods

### Participants

Resting-state fMRI (rs-fMRI) and structural imaging data of 85 individuals with ASD and 163 matched controls from multiple sites of the ABIDE data set (Di Martino et al., 2014) were included in this study for a total of *N* = 248 individuals. Inclusion criteria were: (A) a T1-weighted structural MRI image, (B) a resting-state functional MRI (rs-fMRI) with full cortical coverage, (C) a full-scale IQ > 100, and (D) a mean framewise displacement (FD)(Power et al., 2012) of >0.10 mm. Additionally, individuals for a site were included if a total of at least 7 ASD and 7 control participants met the above inclusion criteria. Demographic information is summarized in Table 1. Details of acquisition, informed consent, site-specific protocols, specific diagnostic criteria for each data set can be found at the ABIDE website http://fcon_1000.projects.nitrc.org/indi/abide/index.html. Institutional Review Board approval was provided by each site.

**Table 1:** Eighty-five individuals with ASD (18.0 yrs, 76 male, IQ = 117, FD = 0.065) and 163 TD children (17.4 yrs, 132 male, IQ = 115.8, FD = 0.064).

**Demographics (Mean ± SD)**	**Controls (*n* = 163)**	**ASD (*n* = 85)**	***P*-value**
Age (years)	17.4 ± 8.0	18.0 ± 10.2	0.84
Sex (% male)	81	89	
IQ	115.8 ± 9.0	117 ± 11.3	0.76
Motion (mm)	0.064 ± 0.02	0.065 ± 0.02	0.70
ADOS-G (score)	NA	10.1 ± 5.4	

### MRI data analysis

#### Structural MRI

T1-weighted structural images were transformed to standard Montreal Neurological Institute (MNI) 2 mm space using the suite of tools available in the FMRIB software library (FSL) 5.0.9 (http://www.fmrib.ox.ac.uk/fsl/). First, skull stripping was performed using the brain extraction tool [BET (Smith, 2002)]. Second, a 12 degrees-of-freedom affine transform from the brain extracted structural image to the MNI 2 mm reference image using FMRIB's linear image registration tool (FLIRT) (Jenkinson et al., 2002). The computed affine transform was applied to the original (non-brain extracted) structural image. Finally, non-linear warping was applied to the linearly registered original structural image using the FMRIB's non-linear image registration tool (FNIRT) (Andersson et al., 2007). Tissue segmentation was performed using FMRIB's automated segmentation tool (FAST) (Zhang et al., 2001). White matter and ventricle masks were created for later use in rs-fMRI nuisance regression. Visual inspection was performed at each stage for each individual to ensure successful brain extraction, tissue segmentation, and normalization.

#### Resting-state functional MRI (rs-fMRI)

The rs-fMRI data were pre-processed as follows. First, correction for rigid body head motion was conducted using motion correction FLIRT (MCFLIRT) (Jenkinson et al., 2002) (default parameters, with final sinc interpolation). Second, an individual's mean rs-fMRI image was aligned with their structural image via a 7 degree-of-freedom affine registration using FLIRT, and the transformation was applied to all volumes in the time series. Frames with excessive motion were identified and scrubbed (Power et al., 2012) if the framewise displacement exceeded 0.3 mm. Individuals with >10% of their frames flagged for scrubbing were excluded. The mean framewise displacement of controls (FD = 0.064) was not significantly different (*W* = 7,135, *p* = 0.74; Table 1) compared to ASD participants (FD = 0.065) as determined by the Wilcoxon rank-sum test. The time series was band-pass filtered removing >0.1 and < 0.01 Hz. Lastly, voxel times series were linear detrended, and reduction of spurious variance was implemented by linear regression of nuisance waveforms derived from head motion (including motion derivatives) and ROI extracted time series in white matter, cerebrospinal fluid (CSF), and global signal. White matter and CSF time series were obtained similar to Chang and Glover (2009) by reverse-normalizing 6 mm spheres at MNI coordinates (26, −12, 35) and (19, −33, 18), respectively, to the native space of each individual. Individual specific white matter and ventricle masks were used to ensure no signal of interest in gray matter was included. Spatially smoothing was performed at the end with a 7 mm FWHM Gaussian filter.

### Functional connectivity principal components analysis

An intra- and inter-network-wise method for analyzing distributed connectivity patterns was employed. Our analyses focused on a putative set of 264 functional regions-of-interest (ROIs) previously organized into 11 RSNs (Power et al., 2011). ROIs were defined as 10 mm diameter spheres whose center coordinates are given in MNI atlas space (Power et al., 2011). For each individual, we computed a 264 × 264 FC matrix by: (i) MNI atlas transformation of the pre-processed functional data, (ii) computation of the mean voxel time series within each ROI, (iii) and computation of the pairwise correlation between all ROI time series.

Data reduction was performed in two steps to isolate a metric of distributed FC changes. First, using each ROI's RSN designation (Power et al., 2011), we computed the average intra- and inter-network correlation for each RSN yielding a reduced 11 × 11 matrix for each individual. The 11 intra-network and $\frac{11\times \left(11-1\right)}{2}=55$ inter-network averages (total of 11+55 = 66) were compiled for all *N* = 248 individuals into a single 248 × 66 matrix M. Second, a principal component analysis (PCA) of the matrix M was performed by singular value decomposition (SVD):

(1) $$\begin{array}{r} \text{M}=\text{UA}{\text{V}}^{\text{T}}. \end{array}$$

PCA is a simple eigenvector-based multivariate analysis that reveals the internal data structure in a way that best explains its variance. A single PCA including both control and ASD individuals provides a set of components common to both groups. This avoids the latent root and vector problem (Krzanowski, 1979) that occurs when separate PCAs are performed for each group. The principal components *c*_*n*_ = *UA* were obtained by projection of the RSN averages onto the principal vectors *V*. The primary component, *c*_1_, was selected. *c*_1_-values vary along the primary vector *V*_1_. Variation along this vector explained 29% of the inter-individual RSN variance.

### Wavelet-based regularity analysis

We computed the entropy, H, of the mean rs-fMRI time series for the same 264 ROIs used in the FC analysis using a previously developed wavelet-based regularity analysis (Smith et al., 2015). This approach is sensitive to, in addition to any non-linear structure, the presence of intrinsic non-stationary processes (i.e., how variable the moments of the signal distribution are over time) within the rs-fMRI signal (Chang and Glover, 2009). Non-stationary structure is preserved with high fidelity across multiple scales using the SWT using the WaveLab850 toolbox (Buckheit et al., 2005). The time series noise level is estimated from the highest frequency subband using wavelet-based de-noising schemes (Donoho and Johnstone, 1994; Donoho, 1995; Chang et al., 2000) and used to tune sensitivity to the entropy of the intrinsic signal. The regularity with which rs-fMRI signal patterns recur is measured with Sample Entropy (Pincus, 1991):

(2) $$\begin{array}{r} \text{H}\left(\text{m},\text{ r},{\text{N}}_{\text{m}}\right)=\;-\log\left(\frac{C^{m+1}\left(\text{r}\right)}{C^{m}\left(\text{r}\right)}\right), \end{array}$$

where recurrence probability of m-length subsequences within a tolerance distance r is given by:

(3) $$\begin{array}{r} {\text{C}}^{\text{m}}\left(r\right)=\;\frac{1}{2}\overset{N_{m}}{\underset{q,p\neq q}{\sum }}\frac{\Theta \left(r\right)}{N_{m}\left(N_{m}-1\right)}. \end{array}$$

*N*_*m*_ is the number of subsequences, Θ is the Heaviside function, and *r* = *r*_0_σ+*t* is the distance threshold for pattern similarity that depends on a scaling *r*_0_ of the time series standard deviation σ and a scale-dependent threshold *t* based on the BayesShrink approach (Chang et al., 2000). Patterns were constructed from time-delayed points to account for the serial correlations present in rs-fMRI data. Pattern lengths were kept small to increase the total number of patterns and improve the statistical power. In this study, patterns of length m(+1) = 1(2) were compared using a distance threshold of *r*_0_ = 0.2. The distance threshold, *r*_0_, was selected using a procedure described previously (Smith et al., 2015). The entropy was computed for a range of thresholds, 0.1–0.3 with 0.05 increments. The *r*_0_-value the maximized the range of observed entropy values across all individuals was selected. The mean entropy across two scales (0.031–0.063 and 0.063–0.13 Hz) for each of the 11 RSNs was obtained for each individual. The dyadic wavelet scales are based on the number of time points. Here, the scales most sensitive to the 0.01–0.10 Hz frequency band, where most slow-wave neuronal activity occurs, were selected.

Patterns containing one or more flagged frames were removed from consideration. Specifically, a binary time series for each individual equal in length to the rs-fMRI frames. Time points equaled one if a frame was flagged for excessive motion. A SWT was applied to this binary series. For each scale, m-length patterns were formed using the same parameters to form patterns for the rs-fMRI series. If any value in these patterns equal one, then the corresponding rs-fMRI pattern is removed from the wavelet-base regularity analysis.

### Multilinear regression model

ASD and controls were pooled together and a multilinear regression model was used to evaluate the relationship between *c*_1_ (FC PCA scores) and RSN entropies, *H*. Specifically, we modeled *c*_1_ as:

(4) $$\begin{array}{r} c_{1}=X_{H}\beta +\varepsilon , \end{array}$$

where *X*_*H*_ is the 248 × 11 matrix of network entropies for the 11 RSNs for all 248 individuals (both ASD and control), β are the model coefficients to be estimated, and ε are the residuals to be minimized. Importantly, no information about group membership (i.e., ASD or control) has been explicitly passed to the model.

Elastic net regularization was performed to avoid overfitting using the “glmnet” package (Friedman et al., 2010) within the R statistical computing language (R Core Team, 2017). Elastic net regularization is a common machine learning approach to building linear models that combines L1 (lasso; Tibshirani, 1996) and L2 (ridge; Tikhonov et al., 1995) regularization. L1 regularization tends to produce sparse solutions by selecting predictors strongly correlated with the outcome and zeroing out the remaining. L2 regularization is suited to deal with high collinearity among predictors. Estimated coefficients, $\hat{\beta }$, from elastic net regularization are formulated as:

(5) $$\begin{array}{r} \hat{\beta }=\underset{\beta }{\min}\left(||c_{1}-X_{H}\beta |{|}^{2}+\frac{\lambda }{2}\left[\left(1-\alpha \right)||\beta |{|}_{2}^{2}+2\alpha ||\beta |{|}_{1}\right]\right) \end{array}$$

where λ is a model complexity parameter, and α is a tradeoff between L1 (α = 1) and L2 (α = 0) regularization. $\hat{\beta }$values represent the importance of certain RSN entropies over others. Model validation was performed using 10-fold cross validation. A grid search for the minimum mean squared error (MSE) was performed across λ and αvalues.

### Statistical analyses

FC PCA score and entropy model distributions for ASD and control individuals were compared using the Wilcoxon rank-sum test. A *post-hoc* linear regression analysis was used to test for an interaction of entropy model estimates, $c_{1H}=X_{H}\hat{\beta }$, by group (i.e., ASD vs. controls) in predicting *c*_1_: *c*_1_ = γ_0_+γ_1_*G*+γ_2_*c*_1*H*_+γ_3_*Gc*_1*H*_+ε_*r*_. Here γ_*i*_ are the regression coefficients, *G* is a binary variable representing ASD individuals or controls, and ε_*r*_ are the regression residuals. The regression coefficient γ_3_ measures the entropy model by group interaction and characterizes the relative model performance between groups. The associations between *c*_1_ and *c*_1*H*_ with the individuals' ADOS-G severity scores (Lord et al., 2000) (for individuals with available scores) were computed using a Pearson correlation. The mean age difference between ASD and controls was 0.6 years, and not statistically significant (*W* = 6,816, *p* = 0.66; Table 1). As such, age was not included as a regressor to avoid loss of statistical power in detecting entropy related group differences.

## Results

### Imbalance in functional connectivity

**Figure 1:**
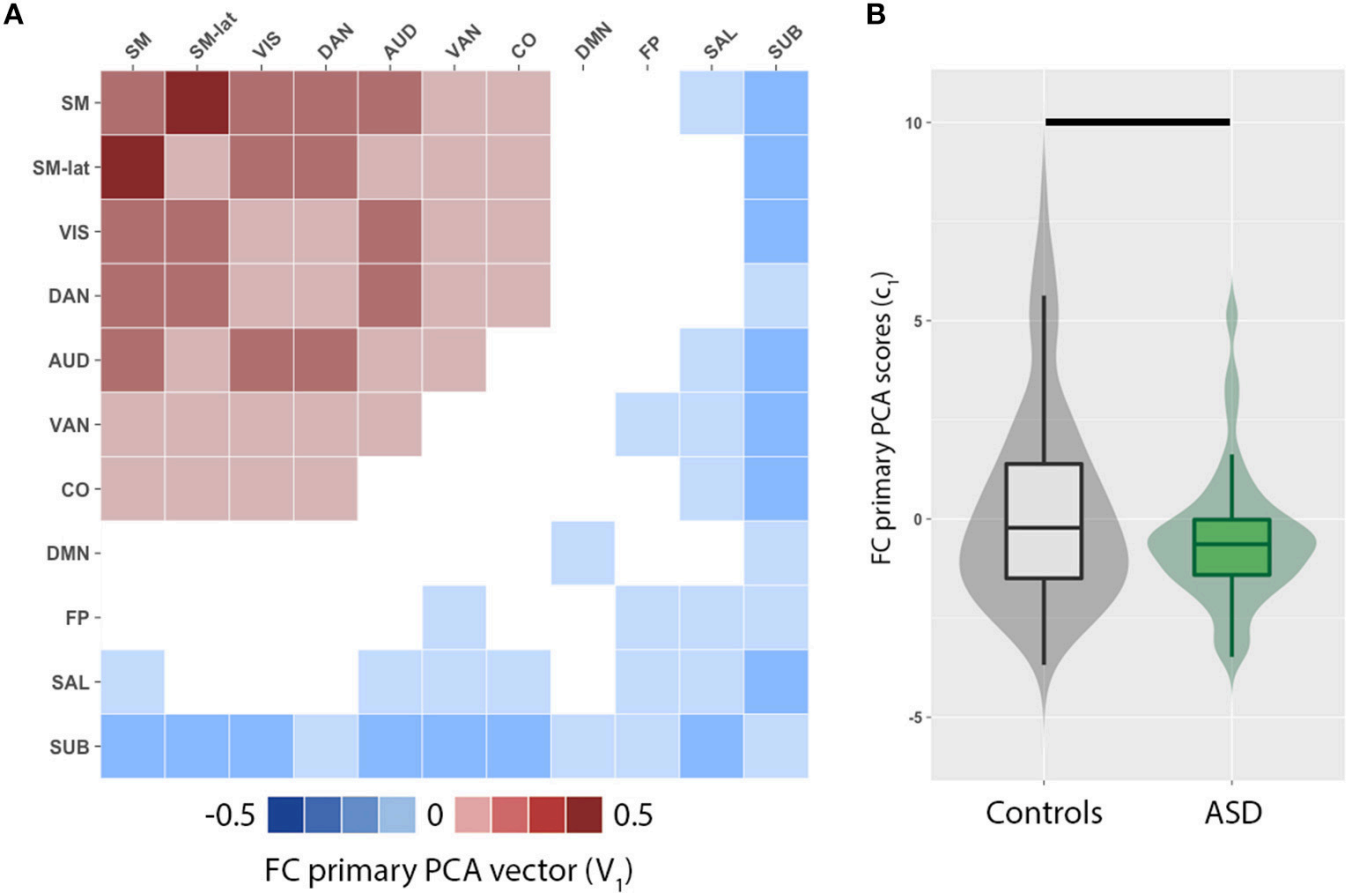
Principal component analysis (PCA) reveals imbalance in functional connectivity (FC) across cortical and subcortical resting-state networks (RSN). **(A)** Primary PCA vector, V_1_, is positively weighted by the intra- and inter-network FC among several RSNs including the sensorimotor (SM, SM-lat), visual (VIS), auditory (AUD), dorsal attention (DAN), ventral attention (VAN), and cingulo-opercular (CO). Conversely, the intra- and inter-network FC of the default mode (DMN), salience (SAL), fronto-parietal (FP), and subcortical (SUB) RSNs are negatively weighted. **(B)** Violin and box plots of the primary PCA component score distributions for controls (gray) and individuals with autism spectrum disorders (ASD; green). Horizontal black line denotes significant difference (*p* = 0.002).

We observed a distributed set of intra- and inter-network FC. The brain networks that exhibit the most inter-individual variation were evaluated by principal component analysis of functional connectivity matrices for ASD and control groups. The primary PCA vector *V*_1_ (Figure 1A) is positively weighted by the intra- and inter-network FC among several RSNs including the sensorimotor (SM, SM-lat), visual (VIS), auditory (AUD), dorsal attention (DAN), ventral attention (VAN), and cingulo-opercular (CO). Conversely, the intra- and inter-network FC of the default mode (DMN), salience (SAL), fronto-parietal (FP), and subcortical (SUB) RSNs were negatively weighted. FC PCA scores, *c*_1_, significantly differed between ASD and control groups (Figure 1B; *W* = 5,846, *p* = 0.04).

### Imbalance in brain entropy

**Figure 2:**
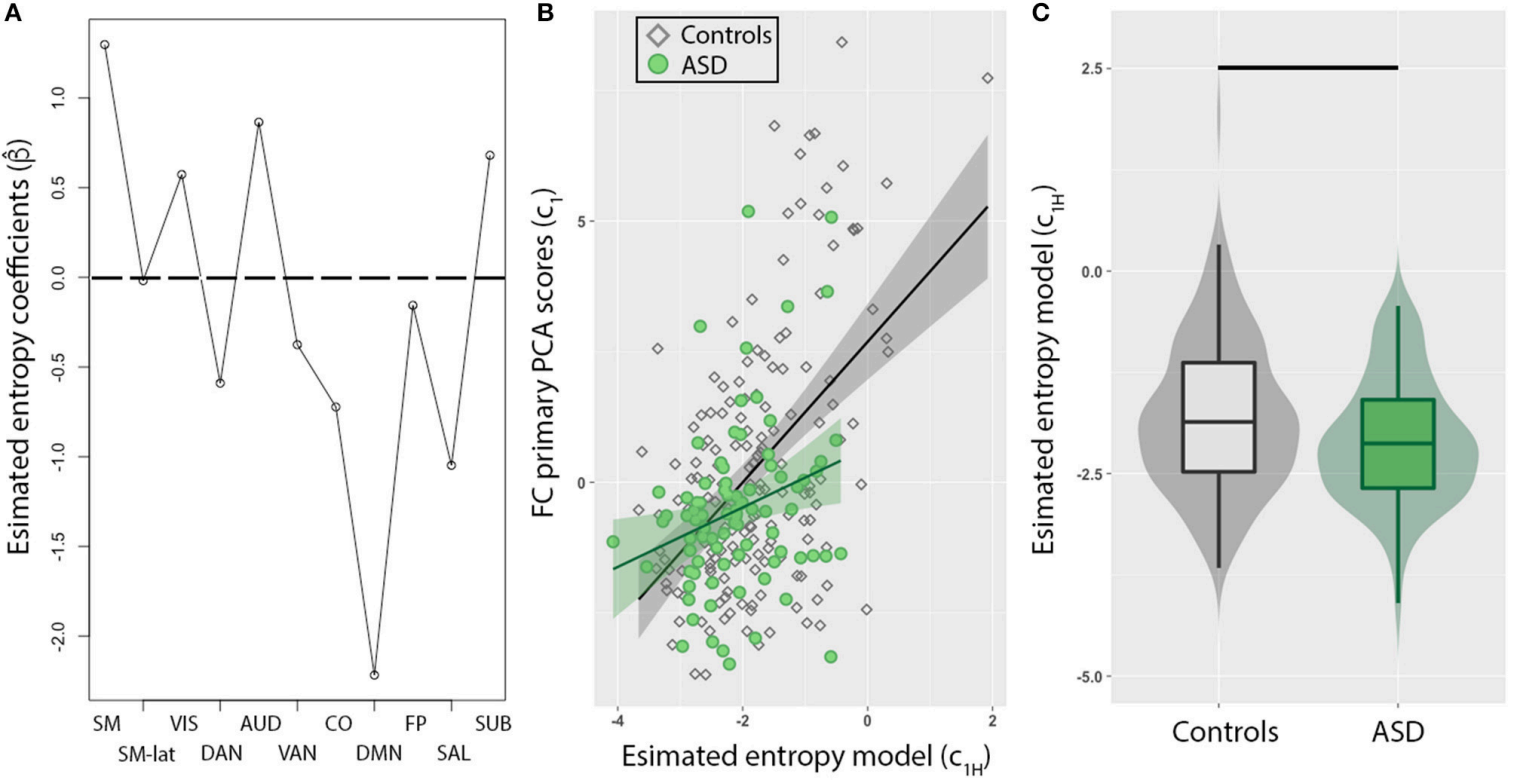
Resting-state network (RSN) entropy is a stronger predictor of functional connectivity (FC) for TD compared to ASD. **(A)** Model coefficients determined from elastic net method show sensory networks (SM, SM-lat, VIS, AUD, SUB) are positively weighted, while higher order cognitive networks (DMN, SAL, CO, DAN, VAN, FP) are negatively weighted. **(B)** Scatter plot showing the relationship between FC (PCA primary component projections) and entropy model for control (diamonds) and ASD (circles) individuals. The model was significantly weaker in predicting FC in ASD compared to controls (*p* = 0.02). **(C)** Box plot of entropy model distributions for control (gray) and ASD (green) groups. Horizontal black line denotes significant difference (*p* = 0.01).

To evaluate whether this imbalance in intra- and inter-network FC in ASD individuals corresponds to dynamical changes, the mean entropy for each of the 11 RSNs were included as predictors to model FC PCA scores (*c*_1_) of all individuals. First, we observed that a combination of most RSNs (Figure 2A) reliably predicted *c*_1_. The minimum MSE computed from a 10-fold cross validation was 11.4 ± 7.2% of *c*_1_ variance, and was observed for α = 0.15. The DMN exhibited the strongest weighting, but interestingly, estimated model coefficients ($\hat{\beta }$), for sensory networks (SM, SM-lat, VIS, AUD, SUB) were positively weighted, while higher order cognitive networks (DMN, SAL, CO, DAN, VAN, FP) were negatively weighted. Second, in a *post-hoc* linear regression analysis that included binary variable representing group (i.e., ASD vs. controls), we observed an interaction of group with *c*_1*H*_ in predicting *c*_1_ (Figure 2B; γ_3_ = 0.82, *t* = 2.3, standard error = 0.35, *p* = 0.02). Lastly, we observed *c*_1H_ were significantly different for ASD compared to controls (Figure 2C; *W* = 5,628, *p* = 0.02).

### Severity score association

**Figure 3:**
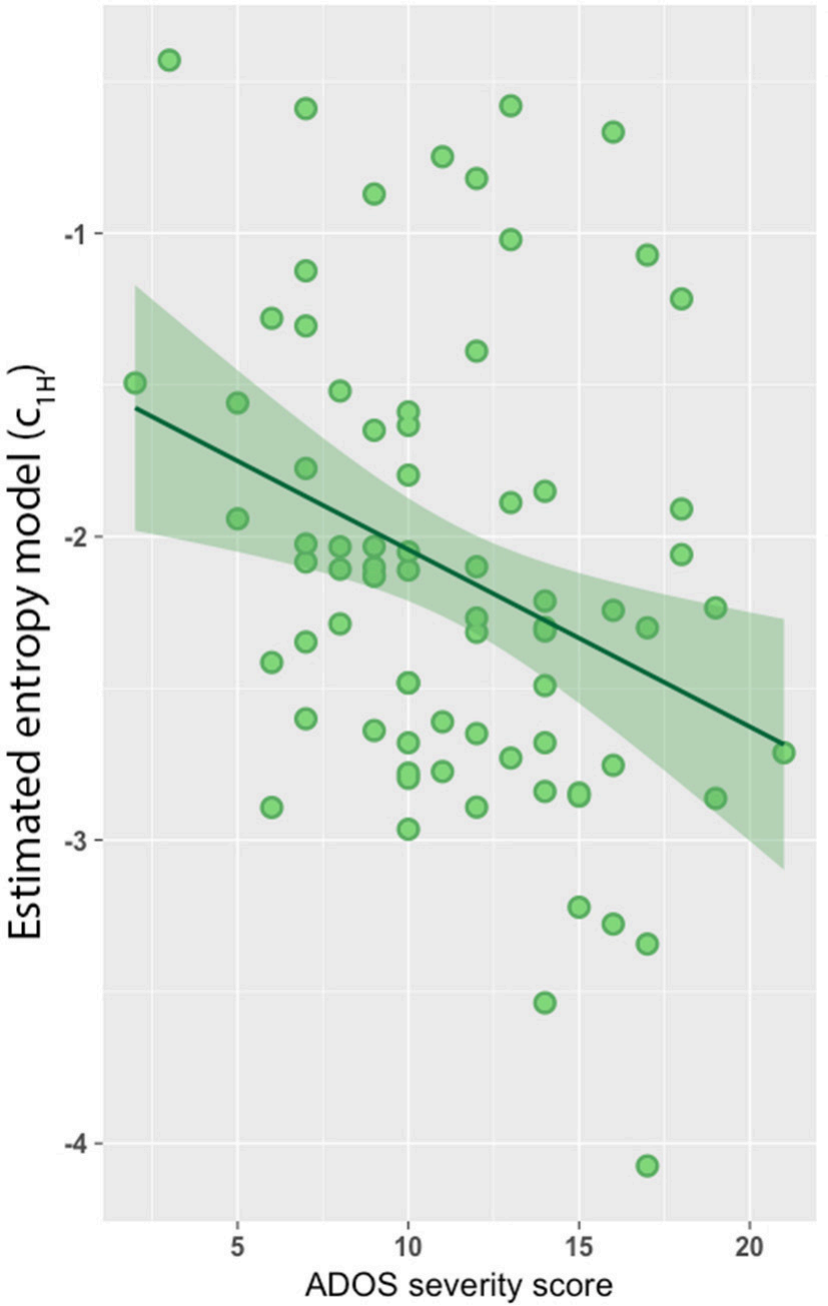
Entropy model predicts autism diagnostic observation schedule-generic (ADOS-G) severity score. Entropy model was negatively associated with ADOS-G severity scores (*r* = −0.31, *p* = 0.003).

Lastly, we found a significant negative association between the estimated model predictors (*c*_1*H*_) and severity scores based on the Autism Diagnostic Observation Schedule-Generic (ADOS-G; Figure 3; *r* = −0.31, *p* = 0.003). However, no association was observed between *c*_1_ and ADOS-G severity scores.

## Discussion

Our findings revealed distributed alterations in FC across multiple RSNs in ASD individuals. Alterations in FC were characterized by negatively weighted sensory and positively weighted cognitive RSNs, suggesting an imbalance of intra- and inter-network FC in ASD. Linear modeling of these alterations in FC revealed a significant association with alterations in brain dynamics, as measured by the time series entropy of multiple RSNs. We observed the observed FC imbalance in ASD individuals was mirrored by a similar imbalance in brain dynamics. Specifically, alterations in brain dynamics were characterized by positively weighted sensory and negatively weighted cognitive RSNs. Alterations in the brain dynamics were further associated with level of symptom severity in individuals with ASD.

Our results provide insight into the impact that ASD has on the intra- and inter-network FC balance among several RSNs. Previous studies have reported hypo-connectivity in the VIS (Villalobos et al., 2005), SM (Mostofsky et al., 2009), and DAN/VAN (Belmonte et al., 2010) networks. Conversely, hyper-connectivity of the salience (Uddin et al., 2013) and subcortical (Padmanabhan et al., 2013; Jann et al., 2015) networks have also been reported. Consistent with these reports, we find imbalanced FC may be a whole-brain phenomenon distributed across multiple RSNs. Further, the imbalanced FC largely discriminated sensory from cognitive networks. Sensory networks primarily develop early during childhood while cognitive networks continue to develop into early adulthood (Somerville et al., 2010; Petanjek et al., 2011). Altered segregation of cognitive networks (as indexed by stronger inter-network connectivity) may reflect the atypical developmental trajectories (e.g., delayed or incomplete pruning process) seen in ASD (Penzes et al., 2011).

There is rapidly growing literature on the relationship between FC and brain dynamics (Hutchison et al., 2013a,b; Allen et al., 2014; Laumann et al., 2017). Here we found that, when taken together, the dynamics of 11 RSNs reliably predicted their engagement of distributed pattern of FC. The strongest contributors to the entropy model were the DMN, SM, and SAL networks. We note, the SM and SAL contributions to both the primary PCA vector and the entropy model were strong, and in both cases opposing each other. This suggests that changes in network dynamics largely follow local FC changes. This is consistent with histological studies reporting disorganized pyramidal cells, consistent with focal cortical dysplasia, extend across many cortical columns in such a fashion that impedes coordinated signaling to other regions in ASD (Casanova, 2007; Schmitz and Rezaie, 2008; Mosconi et al., 2009; Casanova et al., 2013). Conversely, the DMN was a small contributor to the primary PCA vector, but was the largest contributor to the entropy model. This may reflect the tremendous heterogeneity that characterizes ASD (Courchesne et al., 2011). Specifically, both hypo- and hyper-connectivity have been reported within the DMN in individuals with ASD (Raichle and MacLeod, 2001), suggesting these opposing effects may have averaged each other out.

Overall, our results indicate FC and entropy provide complementary information regarding the spatiotemporal organization of the brain. Similar to FC, the entropy model discriminated sensory from cognitive networks. Interestingly our entropy model—rather than FC—was significantly associated with ASD symptom severity. Specifically, the time series signals in the negatively weighted cognitive networks (e.g., DMN, SAL) become less repetitive with increasing symptom severity, suggesting increased excitatory behavior. Conversely, the time series signals in the positively weighted sensory networks (e.g., SM, SUB) become more repetitive with increasing symptom severity. This may suggest increased inhibitory signaling associated with repetitive behaviors in ASD (Lombardo et al., 2016). Taken together, these findings point to entropy as a sensitive measure of the hypothesized excitation and inhibition imbalance underlying ASD behavioral deficits (Rubenstein and Merzenich, 2003; Chao et al., 2010; Vattikuti and Chow, 2010; Yizhar et al., 2011) and may serve as a potential biomarker.

## Ethics statement

This study was carried out in accordance with the recommendations of each sites Institutional Review Board. The protocol was approved by each sites Institutional Review Board. All subjects gave written informed consent in accordance with the Declaration of Helsinki.

## Author contributions

RS, KJ, MD, and DW contributed to the conceptualization of this paper. RS contributed data analysis. RS, KJ, MD, and DW contributed to the drafting of the manuscript.

### Conflict of interest statement

The authors declare that the research was conducted in the absence of any commercial or financial relationships that could be construed as a potential conflict of interest. The reviewer SA and handling editor declared their shared affiliation at time of review.
